# Survey of Tyrosine Kinase Signaling Reveals ROS Kinase Fusions in Human Cholangiocarcinoma

**DOI:** 10.1371/journal.pone.0015640

**Published:** 2011-01-06

**Authors:** Ting-Lei Gu, Xiaxing Deng, Feizhou Huang, Meghan Tucker, Katherine Crosby, Victoria Rimkunas, Yi Wang, Gang Deng, Lei Zhu, Zhiping Tan, Yerong Hu, Chunlin Wu, Julie Nardone, Joan MacNeill, Jianmin Ren, Cynthia Reeves, Gregory Innocenti, Brett Norris, Jin Yuan, Jian Yu, Herbert Haack, Baiyong Shen, Chenghong Peng, Hongwei Li, Xinmin Zhou, Xunyang Liu, John Rush, Michael J. Comb

**Affiliations:** 1 Cell Signaling Technology, Danvers, Massachusetts, United States of America; 2 Center of Organ Transplantation, Ruijin Hospital, Shanghai Jiao Tong University School of Medicine, Shanghai, China; 3 Department of General Surgery, The Third Xiangya Hospital, Central South University, Changsha, China; 4 Department of Cardiothoracic Surgery, The Second Xiangya Hospital, Central South University, Changsha, China; Cedars-Sinai Medical Center, Gene Therapeutics Research Institute, United States of America

## Abstract

Cholangiocarcinoma, also known as bile duct cancer, is the second most common primary hepatic carcinoma with a median survival of less than 2 years. The molecular mechanisms underlying the development of this disease are not clear. To survey activated tyrosine kinases signaling in cholangiocarcinoma, we employed immunoaffinity profiling coupled to mass spectrometry and identified DDR1, EPHA2, EGFR, and ROS tyrosine kinases, along with over 1,000 tyrosine phosphorylation sites from about 750 different proteins in primary cholangiocarcinoma patients. Furthermore, we confirmed the presence of ROS kinase fusions in 8.7% (2 out of 23) of cholangiocarcinoma patients. Expression of the ROS fusions in 3T3 cells confers transforming ability both *in vitro* and *in vivo*, and is responsive to its kinase inhibitor. Our data demonstrate that ROS kinase is a promising candidate for a therapeutic target and for a diagnostic molecular marker in cholangiocarcinoma. The identification of ROS tyrosine kinase fusions in cholangiocarcinoma, along with the presence of other ROS kinase fusions in lung cancer and glioblastoma, suggests that a more broadly based screen for activated ROS kinase in cancer is warranted.

## Introduction

Despite major efforts to improve diagnosis and treatment of liver cancer, the five-year survival rate of individuals with this disease is very poor, marking this malignancy as one of the most lethal cancers[1]. Primary liver cancer comprises histologically distinct hepatic neoplasms. The two most common types of liver cancer are hepatocellular carcinoma (HCC), accounting for 80% of all cases, and cholangiocarcinoma (CCA, or bile duct cancer), representing 10–15% of hepatobiliary neoplasms [2], [3]. While chronic hepatitis B and C infection, alcohol consumption, and toxins are risk factors associated with HCC, little is known about the molecular pathogenesis of cholangiocarcinoma[4]. There are over 90 annotated tyrosine kinases in the human genome that are important regulators of intracellular signal transduction pathways mediating cellular proliferation, survival, and development [5]. The activity of these kinases is normally tightly regulated, and constitutive activation of tyrosine kinases by acquired somatic mutation contributes to oncogenic transformation in many cancers [6].

To facilitate the identification of tyrosine kinases and phosphorylation events involved in the pathogenesis of cholangiocarcinoma, we applied a strategy based on immunoaffinity purification of tyrosine phosphorylated peptides followed by LC-MS/MS based identification [7], [8], [9]. Using this phosphoproteomic approach, we broadly surveyed tyrosine kinase signaling in primary cholangiocarcinomas, and identified activated ROS kinase not previously known to play a role in cholangiocarcinoma. Upon further biochemical and functional analysis, we confirmed the oncogenic property of ROS kinase fusions. This is the first report of chromosomal translocation involving a tyrosine kinase in cholangiocarcinoma, and provides new insights into signaling pathways and therapeutic targets in this disease.

## Results

### Profiling of phosphotyrosine signaling in cholangiocarcinoma by LC-MS/MS mass spectrometry

**Figure 1:**
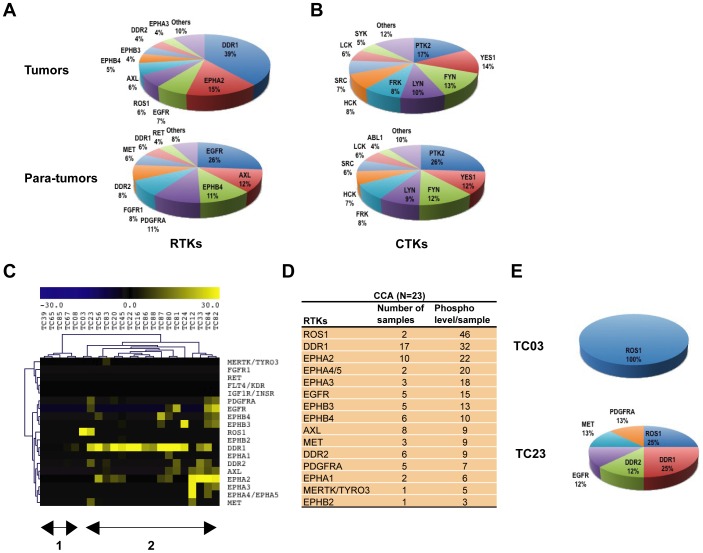
Identification of aberrantly phosphorylated tyrosine kinases in cholangiocarcinoma. (**A**) and (**B**) Distribution of receptor tyrosine kinases (RTKs) and non-receptor tyrosine kinases (CTKs) in tumors and matching para-tumor tissues. The total number of spectral counts of each RTK/CTK is normalized against total number of phosphopeptides of GSK3A (100) in each sample, then the sum of the normalized number of each RTK/CTK as fractions of the total are shown. See Table S2. (**C**) Receptor tyrosine kinase (RTK) profiles from 23 CCA samples revealed heterogeneous tyrosine kinase activities in CCA. Average RTK signals from 20 normalized para-tumor tissues were subtracted from each CCA sample. The yellow color represents kinases aberrantly phosphorylated in CCA, and the blue color represents kinases under phosphorylated in tumor. ‘TC’ for cholangiocarcinoma tumor samples. (**D**) Ranking of RTK phosphorylation in CCA. Phospho level/sample was derived from average of normalized phosphopeptide spectra of each RTK from tumor samples showing positive signal of this RTK. (**E**) Distribution of RTKs in two cholangiocarcinoma samples (TC03 and TC23). RTK values were presented as fractions of the total RTK values from each sample.

To survey protein tyrosine phosphorylation in cholangiocarcinoma (CCA), we applied an immunoaffinity phosphoproteomic approach [9]. Resected primary CCA were homogenized and digested with trypsin, phosphopeptides were immunoprecipitated with phosphotyrosine antibody (pY-100), and analyzed by LC-MS/MS mass spectrometry [8], [9]. Matching para-tumor tissues of similar size were also included in the study. Table S1 shows phosphotyrosine profiles from 23 primary CCA and 20 para-tumor tissues. About 1053 tyrosine phosphorylation sites were identified on 746 different proteins by high resolution, high accuracy MS, with the global false positive rate to be less than 5.0%. This study significantly extended our knowledge of tyrosine kinase signaling in cholangiocarcinoma, and these data have been deposited in PhosphoSitePlus™ (www.phosphosite.org), a freely accessible database for phosphorylation and other posttranslational modifications. First, we compared the receptor tyrosine kinase (RTK) phosphorylation profile between tumors and para-tumor tissues. While tumors show high levels of DDR1, EphA2, EGFR, and ROS1 tyrosine kinase phosphorylation, para-tumor tissues showed the highest level of tyrosine phosphorylation in EGFR, AXL, EPHB4, and PDGFRA. Of note, we also observed the presence of MET kinase activity in para-tumor tissues, consistent with the requirement of EGFR and hepatocyte growth factor/c-met signaling pathways for normal hepatocyte development, as well as liver regeneration [10], [11] (Figure 1A). On the other hand, PTK2 (FAK) and SRC-family kinases make up the majority of cytoplasmic tyrosine kinase (CTK) phosphorylation in these two groups (Figure 1B).

Cholangiocarcinoma is a heterogeneous disease, individual patient may have distinct tyrosine kinase profile. To identify aberrant receptor tyrosine kinases (RTKs) signals in individual CCA, the number of phosphopeptides per RTK was normalized against total number of phosphopeptides of GSK3A (100) from each sample (Table S2)[8], then average RTK signals from 20 para-tumor tissues were subtracted from each CCA tumor (N = 23). Unsupervised hierarchical clustering revealed the presence of two groups, tumors expressing little or no RTKs activity (group 1, N1 = 5, 22%), and tumors expressing kinases, such as DDR1, EPHA2, and ROS1 (group 2, N2 = 18, 78%) (Figure 1C). To better understand aberrant tyrosine kinases activities in CCA, we applied a ranking method developed by Rikova et al [8]. As shown in Figure 1D, ROS1, DDR1, and EPHA2 are the top ranked kinases identified in CCA. Furthermore, phosphopeptides from ROS kinase were identified in two tumors (TC03 and TC23), but not in the corresponding para-tumor tissues (Table S1, NC23 and NC03), and presented as the major tyrosine kinase activities in these two samples (Figure 1E).

**Figure 2:**
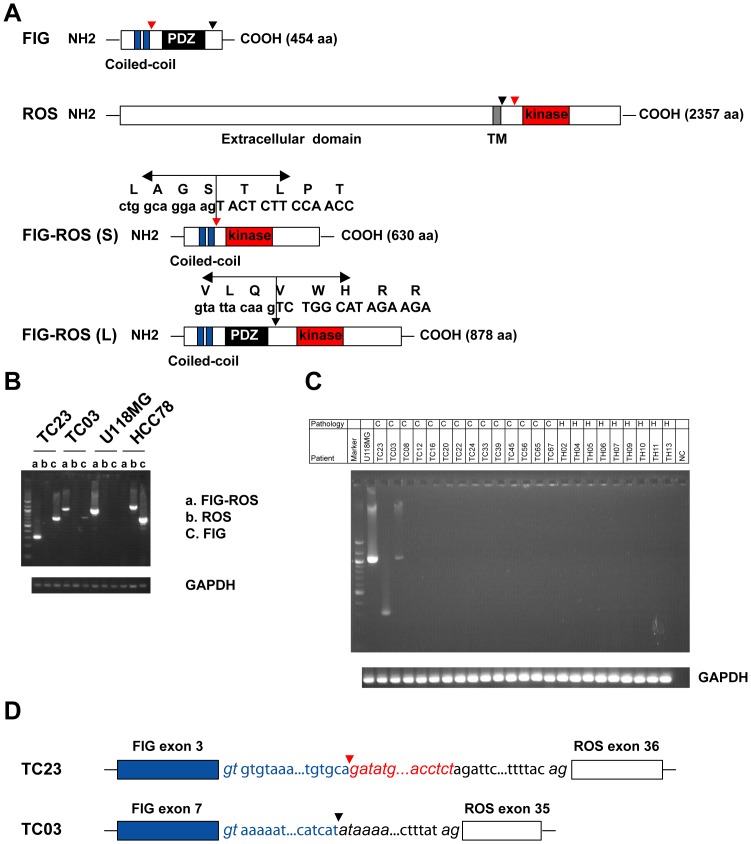
Identification of ROS kinase fusions in CCA patients. (**A**) Schematic diagram shows the Fused in Glioblastoma (FIG), ROS and FIG-ROS proteins. The position of the breakpoint is indicated by arrowhead (red or black). PDZ stands for PDZ domain, a protein-interaction domain; TM for transmembrane. Blue boxes refer to the two coiled-coil domains. The amino acid and DNA sequence from junction of the ROS fusions are listed. FIG-ROS (L) and FIG-ROS (S) refer to the long form and the short form of ROS fusions, respectively. (**B**) Expression of FIG and ROS mRNA in FIG-ROS positive cholangiocarcinoma patients. a. primer pairs FIG-F2 and ROS-GSP3.1 for FIG-ROS. b. primer pairs ROS-Ex31F and ROS-GSP2 for ROS. c. primer pairs FIG-F3 and FIG-R8 for FIG. U118MG and HCC78 were used as controls. (**C**) RT-PCR reaction identified a fusion of *FIG* to *ROS* in cDNA from two cholangiocarcinoma patients. GAPDH was used as a control. cDNA from U118MG cell line was included as a positive control. ‘C’ for cholangiocarcinoma, and ‘H’ for hepatocellular carcinoma. (**D**) Genomic breakpoints of *FIG* and *ROS* fusion gene for each patient. *FIG* intron sequences are shown in blue, and *ROS* intron sequences are shown in red or black. For TC23, the intron sequences between FIG-ROS fusion gene are composed of 1–822 base pair (bp) from intron 3 of FIG, antiparallel sequence of 620–656 bp derived from intron 35 of ROS (shown in red), and 666–1228 bp from intron 35 of ROS. For TC03, the intron sequences between FIG-ROS fusion gene consist of 1–2402 bp from intron 7 of FIG and 2317–2937 bp from intron 34 of ROS. Splice donor acceptor sites are shown in Italics.

DNA sequencing analysis did not detect any mutations in the kinase domains of ROS (data not shown). While the full length ROS protein has a molecular weight of 258 kDa, a truncated form of ROS protein (60–80 kDa) was detected by Western blot analysis from one of the ROS positive primary tumor (TC23) (Figure S1A). To elucidate the molecular mechanisms leading to truncation and activation of ROS kinase in CCA, we performed 5′ rapid amplification of cDNA ends (5′ RACE) on RNA from two ROS positive patient samples. Sequence analysis of the resulting product showed that the kinase domain of ROS was fused in-frame to the Fused in Glioblastoma (*FIG*) gene. Two different forms of FIG-ROS fusion were identified (Figure 2A). In sample TC23, exon 3 of FIG was fused to exon 36 of ROS. The fusion protein, named FIG-ROS(S), combines the amino terminal 209 amino acids of FIG with the carboxyl terminal 421 amino acids of ROS. In sample TC03, exon 7 of FIG was fused to exon 35 of ROS. The fusion protein, FIG-ROS(L), combines the first 412 amino acids of FIG with the carboxyl terminal 466 amino acids of ROS. It is the same FIG-ROS fusion protein previously identified in a glioblastoma cell line U118MG, representing the first case of the same tyrosine kinase fusion involved in two distinct types of cancer. FIG (GOPC), a PDZ domain containing Golgi protein, plays an important role in intracellular protein trafficking and degradation [12]. Meanwhile, ROS tyrosine kinase is an orphan receptor whose normal expression pattern is tightly spatio-temporally regulated during development [13]. While we did not detect the expression of wild type ROS gene, expression of FIG gene was confirmed in these two samples (Figure 2B), suggesting that expression of FIG-ROS fusion gene contributes to the tyrosine phosphorylation of ROS kinase. U118MG cells, which are known to have homozygous deletion at 6q21 [14], did not express either wild type FIG or ROS gene. HCC78, a non-small cell lung cancer cell line, which contains SLC34A2-ROS fusion [8], expresses both FIG and ROS gene. The fusion product of *FIG* and *ROS* was further confirmed by reverse-transcriptase-PCR (Figure 2C). In addition, we did not detect any FIG-ROS fusions in over 60 hepatocellular carcinoma samples (data not shown). Moreover, genomic PCR was performed to identify the genomic breakpoint for each patient (Figure 2D and Figure S1B). Attempts to amplify the reciprocal fusion genes were unsuccessful (data not shown), indicating that all fusions were the result of a deletion on 6q21 and not of t(6;6). Thus, we identified 2 patients with ROS kinase fusions in 23 CCA, with a frequency of 8.7%.

### FIG-ROS fusions transform NIH3T3 cells both *in vitro* and *in vivo*

**Figure 3:**
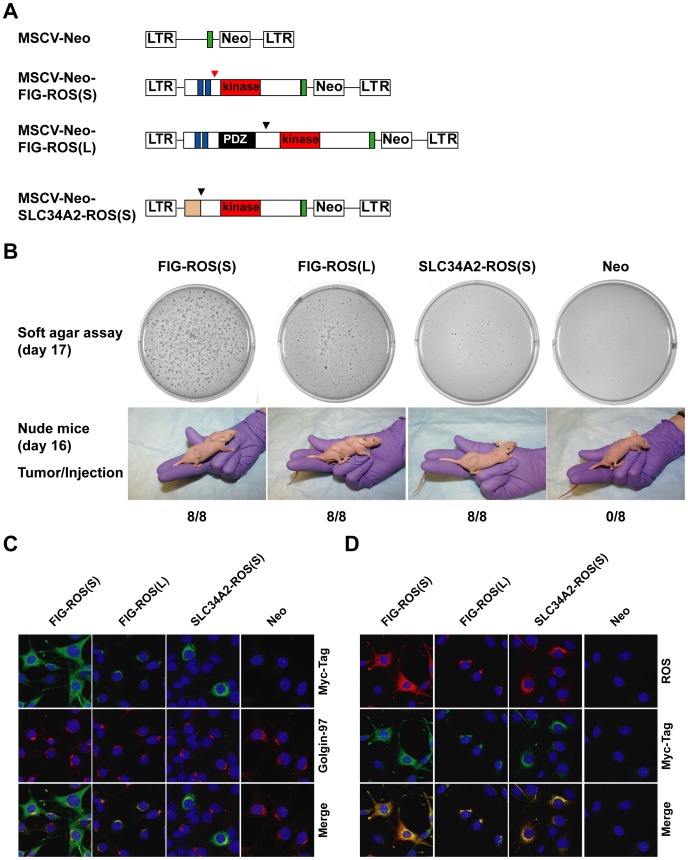
Characterization of FIG-ROS fusions. (**A**) Schematic representations of FIG-ROS used in the study. MSCV denotes murine stem cell virus; Neo for Neomycin; LTR for long terminal repeat; Green box for Myc-Tag. (**B**) Expression vectors for FIG-ROS(S), FIG-ROS(L), and SLC34A2-ROS(S) (or the corresponding empty vector) were introduced into 3T3 cells. Triplicate experiments were performed. The cells from representative experiment were photographed after 17 days of culture (upper panels). The same set of transfected cells was also injected subcutaneously at 2 sites into each nude mice, and tumor formation was examined after 16 days (lower panels). The number of tumors formed after eight injections are indicated. (**C**) and (**D**) ROS fusions display distinct subcellular localization. Shown is indirect immunofluorescence analysis of clonally derived 3T3 cells expressing FIG-ROS(S), FIG-ROS(L), SLC34A2-ROS(S) (or the corresponding empty vector). Cells were fixed and stained with Myc-Tag, Golgin-97(Golgi marker), and ROS antibody. Nuclei are stained with DAPI (blue).

While FIG-ROS(L) was previously reported to be oncogenic both *in vitro* and *in vivo* [14], [15], not much is known about the transforming ability of FIG-ROS(S). To this end, we transfected 3T3 cells with retroviral constructs (C- terminal Myc-Tag) containing FIG-ROS(S), FIG-ROS(L), and SLC34A2-ROS(S), respectively. SLC34A2-ROS(S) was the short form of ROS fusion previously identified in NSCLC [8]. pMSCV vector lacking any ROS fusion cDNA was used as a negative control (Figure 3A). 48 hours after transfection, 3T3 cells were selected for neomycin resistance for 7 days. Western blot analysis showed that both forms of FIG-ROS fusions activate known downstream effectors of ROS, such as STAT3 and AKT (Figure S2A). On the other hand, SLC34A2-ROS(S) has minimal effects on STAT3 and AKT in this system (Figure S2B). To determine whether FIG-ROS(S) can cause anchorage-independent growth of 3T3 cells, stably transfected 3T3 cells were cultured in soft agar for 17 days. As shown in Figure 3B (top panel), FIG-ROS(S) expressing 3T3 cells formed large number of colonies in soft agar, whereas none were observed in the negative control, indicating that FIG-ROS(S) can transform 3T3 cells *in vitro*. Meanwhile, the presence of either FIG-ROS(L) or SLC34A2-ROS(S) also enabled 3T3 cells to form colonies, although the effect was not as significant as that seen with FIG-ROS(S). Thus, it is possible that that FIG-ROS(S) might be a more potent kinase than FIG-ROS(L). To further investigate the transforming ability of FIG-ROS(S) *in vivo*, Immunocompromised nude mice were injected with 1×10^6^ 3T3 cells transduced with retrovirus containing empty vector, FIG-ROS(S), FIG-ROS(L), or SLC34A2-ROS(S). Mice were monitored daily for tumor formation and size, and were sacrificed when tumors reached approximately 1 cm×1 cm. As shown in Figure 3B (bottom panel), two weeks after being injected with 3T3 cells transduced with either FIG-ROS(S), FIG-ROS(L) or SLC34A2-ROS(S), tumor formation was apparent in all the injected nude mice. In contrast, tumors were not found in mice injected with pMSCV vector control 3T3. Thus, we confirmed that, like FIG-ROS(L), FIG-ROS(S) is tumorgenic both *in vitro* and *in vivo*.

The transforming ability of FIG-ROS(L) requires targeting to the Golgi apparatus through the second coiled-coil domain of FIG [16]. To ascertain the subcellular localization of FIG-ROS(S), we performed immunofluorescence assay with 3T3 cells stably transfected with the ROS fusion variants with Myc-tag antibody. As expected, FIG-ROS(L) targets to the Golgi apparatus, and co-localizes with the Golgi marker (golgin-97). To our surprise, the staining pattern of FIG-ROS(S) was cytoplasm, even though it contains the second coiled-coil domain of FIG (Figure 3C), suggesting that the coiled-coil domain of FIG is necessary, but not sufficient to target FIG-ROS(S) to the Golgi apparatus. Interestingly, SLC34A2-ROS(S) was localized to para-nuclei compartment. These results were further confirmed by a ROS antibody (Figure 3D). Thus, different ROS fusions have distinct subcellular localization, suggesting that they may activate different substrates *in vivo*.

### FIG-ROS fusion is a potential therapeutic target

**Figure 4:**
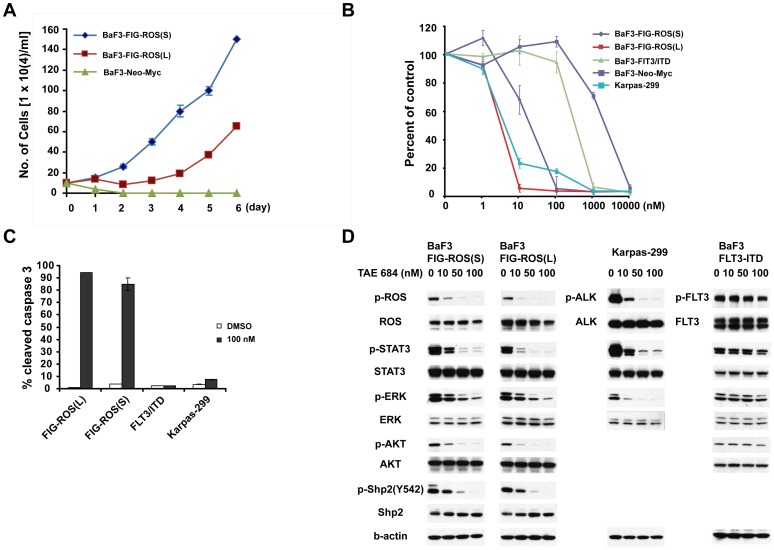
Transformation, inhibition, and signaling properties of the FIG-ROS fusion tyrosine kinase. (**A**) BaF3 cells retrovirally transduced with ROS fusion constructs were grown in the absence of IL-3. (**B**) Dose response graph of TAE684 for BaF3 cells expressing FIG-ROS fusions. BaF3/FLT3-ITD, and Karpas-299 (NPM-ALK) cells were used as controls. Triplicate experiments were performed. (**C**) Treatment with TAE684 increased apoptosis of BaF3 cells expressing FIG-ROS fusions, but not FLT3-ITD as measured by cleaved caspase-3 staining. Representative results from triplicate experiments were included. (**D**) Incubation with different concentrations of TAE684 resulted in decreased phosphorylation of ROS kinase, accompanied by decreased phosphorylation of STAT3, AKT, ERK, and Shp-2.

The oncogenecity of FIG-ROS fusions were further evaluated by their abilities to transform interleukin-3 (IL-3)-dependent murine lymphoid BaF3 cells to cytokine-independent growth. Retroviral transduction of either FIG-ROS(S) or FIG-ROS(L) transformed BaF3 cells to factor independent growth (Figure 4A and Figure S2C), and there is a shorter latency for FIG-ROS(S) to transform BaF3 cells than seen with FIG-ROS(L), indicating that FIG-ROS(S) might be a more potent kinase than FIG-ROS(L) as suggested by previous soft agar assay. To confirm this finding, we performed in vitro kinase assay. While both forms of FIG-ROS fusions showed increased tyrosine kinase activity in vitro as compared to control, FIG-ROS(S) has more than 4 fold higher kinase activity than FIG-ROS(L) (Figure S2D). Since ROS kinase shares high sequence homology with ALK, we evaluated the potential of TAE684 (an ALK inhibitor) to inhibit ROS kinase activity and signaling. Treatment of TAE684 abolished the growth of BaF3 cells expressing either FIG-ROS(S) or FIG-ROS(L) with IC50 of 10 nM and 1.8 nM, respectively (Figure 4B). As expected, NPM-ALK expressing Karpas-299 is sensitive to TAE684 with an IC50 of 4.8 nM, similar to the IC50 previously reported [17]. On the contrary, BaF3 cells expressing FLT3-ITD is not sensitive to TAE684, neither did BaF3 cells expressing empty vector (Neo-Myc). These data confirm that FIG-ROS is the target of TAE684. To understand the biological effects of inhibition of FIG-ROS on the growth and survival of BaF3 cell lines, we performed apoptosis analysis on cells treated with either TAE684 or DMSO. BaF3 FIG-ROS(S), BaF3 FIG-ROS(L), BaF3 FLT3-ITD, and Karpas-299 cells were treated with 100 nM of TAE684 for 48 h and were assessed for induction of apoptosis by flow cytometry analysis. At 48 h after incubation with TAE684, 85–95% of FIG-ROS expressing cells stained positive for cleaved caspase 3 in several independent experiments. In contrast, no increase in the number of cleaved caspase-positive cells was seen in BaF3 cells expressing FLT3-ITD (Figure 4C). Although karpas-299 did not undergo significant apoptosis when treated with TAE684, it was primarily due to cell cycle arrest (data not shown) [17]. To find out whether TAE684 also inhibits signaling downstream of FIG-ROS, FIG-ROS expressing BaF3 cells were treated with either DMSO or increasing concentrations of TAE684 for 3 hours. As demonstrated in Figure 4D, TAE684 inhibited ROS phosphorylation in a dose dependent manner. The impact of FIG-ROS inhibition on its downstream signaling was evaluated by using p-STAT3, p-AKT, p-ERK, and p-Shp2 as surrogate markers for JAK/STAT, PI3K-AKT, RAS/MAPK pathways. Clearly, inhibition of FIG-ROS by TAE684 led to a dose-dependent reduction in phosphorylation of STA3, AKT, ERK, and Shp2 in BaF3 cells. As expected, we observed inhibition of ALK downstream signaling molecules in Karpas-299 cells upon treatment of TAE684. In contrast, we do not see significant changes in the phosphorylation of FLT3 and its downstream signaling intermediates. These results demonstrate that TAE684 inhibits not only FIG-ROS, but also its crucial downstream signaling molecules.

## Discussion

In this study, we surveyed tyrosine kinase signaling events in cholangiocarcinoma using an unbiased phosphoproteomic approach. This approach is a sensitive and reproducible functional strategy to identify activated protein kinases and their phosphorylated substrates without prior knowledge of the signaling networks [7], [8]. Furthermore, in the context where protein tyrosine kinases are known to play an important role in many human cancer genes [6], [18], phosphoproteomic analysis provides a functional screening assay to rapidly identify constitutively activated tyrosine kinases regardless of the molecular mechanism of activation. This analysis generated a deep and broad view of tyrosine kinase activity and downstream signaling networks that were not revealed before.

By following up tyrosine kinase profile in individual patient, we identified activated ROS kinase in cholangiocarcinoma. Elevated ROS expression was also observed in non-small cell lung cancer (NSCLC) and breast cancer [19], [20], [21]. Demethylation of ROS promoter contributes to the elevated expression of ROS kinase in malignant gliomas [22]. Chromosomal rearrangements involving ROS kinase have been reported in glioblastoma and non-small cell lung cancer [8], [14]. Since expression of FIG-ROS in CNS induces glioblastoma formation *in vivo* [15], we speculate that expression of FIG-ROS could develop cholangiocarcinoma *in vivo* as well.

In the present study, we identified aberrant ROS kinase expression in 8.7% cholangiocarcinoma patients. Cholangiocarcinoma is the second most common primary hepatic carcinoma. Advanced cholangiocarcinoma has a median survival of less than 2 years. While the only curative therapy is surgical extirpation or liver transplantation, most patients with cholangiocarcinoma present with advanced stage disease, which is not suitable for surgery [2], [3]. Our data suggest that inhibition of the tyrosine kinase activity of ROS may induce growth inhibition and cell death in BaF3 cells expressing this fusion protein. Thus, specific ROS inhibitors may provide means to treat patients with liver cancer that expresses ROS fusions, for whom effective treatments are rarely available. Since attempts to identify cholangiocarcinoma cell lines containing FIG-ROS fusions were unsuccessful, these FIG-ROS transformed BaF3 cell lines could be used as in vitro models to screen ROS inhibitors. Given that the association of FIG-ROS with both cholangiocarcinoma and glioblastoma, it will be important to examine the association of FIG-ROS and other activated ROS alleles with other types of cancers, as well as in other ethnic groups. By integrating genetic, epigenetic, proteomic, and phosphoproteomic information, we can begin to understand the pathogenesis of cholangiocarcinoma and identify novel therapeutic targets.

## Materials and Methods

### Cell lines and tumors

BaF3 and Karpas-299 cells were obtained from DSMZ (Deutsche Sammlung von Mikroorganismen und Zellkulturen GmbH, Germany). U118 MG, HCC78, and 3T3 cells were purchased from American Type Culture Collection (Manassas, VA). BaF3 cells were maintained in RPMI-1640 medium (Invitrogen) with 10% fetal bovine serum (FBS) (Sigma) and 1.0 ng/ml IL-3 (R&D Systems). Karpas-299 cells were grown in RPMI-1640 with 10% FBS. Other cell lines were grown in DMEM with 10% FBS.

Cholangiocarcinoma tumors (n = 23), as well as matching para-tumor tissues (n = 20) were collected within 15 minutes from surgical resections from patients when sufficient material for PhosphoScan® analysis, RNA, and DNA extractions were available. According to the Edmondson grading system, all tumor samples have differentiation grades II–III. The tumor specimens were collected at RuiJin hospital (Shanghai, China) and Third Xiangya hospital (Changsha, Hunan, China) with written consent from patients. Patient information was not revealed in this study, and the data were analyzed anonymously. Obtaining patient materials were approved by both Ruijin hospital and third Xiangya hospital institutional review board.

### Phosphopeptide immunoprecipitation and analysis by LC-MS/MS Mass Spectrometry

Phosphopeptides were prepared using PhosphoScan® Kit (Cell Signaling Technology). In brief, about 200–500 mg tumor samples were homogenized and lysed in urea buffer, trypsin digested lysates were purified by Sep-pak C_18_ column (Waters). Then, lyophilized peptides were redissolved and immunoaffinity purified with pY-100 antibody. pTyr-containing peptides were concentrated on reverse-phase micro tips. LC-MS/MS analysis was performed with an LTQ Orbitrap Mass Spectrometer (Thermo Fisher Scientific) and a peptide mass accuracy of ±3 ppm was one of the filters used for peptide identification. Details were described previously [8]. In brief, samples were collected with an LTQ – Orbitrap hybrid mass spectrometer, using a top-ten method, a dynamic exclusion repeat count of 1, and a repeat duration of 30 sec. MS spectra were collected in the Orbitrap component of the mass spectrometer and MS/MS spectra was collected in the LTQ. Sequest (Thermo Fisher Scientific) searches were done against the NCBI human database released on July 02, 2009, (containing 37,391 proteins), allowing for tyrosine phosphorylation (Y+80) and oxidized methionine (M+16) as differential modifications. The PeptideProphet probability threshold was chosen to give a false positive rate of 5% for the peptide identifications[23].

### Clustering analysis

For each patient sample, each protein's spectral counts were normalized to those for GSK3A (100). We used the following statistical and computational tools from GenePattern 3.0 software package (Broad Institute of MIT and Harvard) for Comparative Marker Selection; from MultiExperiment Viewer version 4.4 for Hierarchical Clustering (Pearson correlation distance and complete linkage clustering).

### Rapid Amplification of Complementary DNA Ends

RNeasy Mini Kit (Qiagen) was used to extract RNA from human tumor samples. DNA was extracted with the use of DNeasy Tissue Kit (Qiagen). Rapid amplification of cDNA ends was performed with the use of 5′ RACE system (Invitrogen) with primers ROS-GSP1 for cDNA synthesis and ROS-GSP2 and ROS-GSP3.1 for a nested PCR reaction, followed by cloning and sequencing PCR products.

### Transfection, cell proliferation and growth assays

Transfections were carried out using FuGENE 6 (Roche Diagnostics), and retrovirus was harvested at 48 after transfection. BaF3 cells were transduced with retroviral supernatant containing either the MSCV-Neo/FIG-ROS(L) or MSCV-Neo/FIG-ROS(S) vector, and selected for G418 (0.8 mg/ml). IL-3 independent growth was accessed by plating transduced BaF3 cells in IL-3 free medium, after the cells were washed three times in PBS. For dose response curves, cells were incubated for 72 hours in the presence of TAE684 (customer synthesized), and the number of viable cells was determined with the CellTiter 96 AQ_ueous_ One solution cell proliferation assay (Promega). IC_50_ was calculated with the use of OriginPro 6.1 software (OriginLab). The percentage of apoptotic cells at 48 hours was determined by flow cytometric analysis of cleaved caspase-3 (Cell Signaling Technology).

### Immunofluorescence assay

3T3 cells stably transfected with myc tagged FIG-ROS(L), FIG-ROS(S), or empty vector were subjected to immunofluorescence assay according to protocol (Cell Signaling Technology).

### PCR Assay

For RT-PCR, first-strand cDNA was synthesized from 2.5 ug of total RNA with the use of SuperScript™ III first-strand synthesis system (Invitrogen) with oligo (dT)_20_. Then, the *FIG-ROS* fusion gene was amplified with the use of primer pairs FIG-F2 and ROS-GSP3.1. Wild type *FIG* and *ROS* gene was amplified with the use of primer pairs FIG-F3 and FIG-R8, ROS-Ex31F and ROS-GSP2, respectively. For genomic PCR, amplification of the fusion gene was performed with the use of LongRange PCR kit (Qiagen) with primer pairs FIG-F3 and ROS-GSP3.1 for TC23, or FIG-F7 and ROS-GSP4.1 for TC03 and U118MG.

### Primers

The following primers were used:

ROS-GSP1: 5′ACCCTTCTCGGTTCTTCGTTTCCA

ROS-GSP2: 5′TCTGGCGAGTCCAAAGTCTCCAAT

ROS-GSP3.1: 5′CAGCAAGAGACGCAGAGTCAGTTT

FIG-F2: 5′ACTGGTCAAAGTGCTGACTCTGGT

FIG-F3: 5′TTGGATAAGGAACTGGCAGGAAGG

FIG-R8: 5′ACCGTCATCTAGCGGAGTTTCACT

ROS-Ex31F: 5′AGCCAAGGTCCTGCTTATGTCTGT

FIG-F7: 5′ TGTGGCTCCTGAAGTGGATTCTGA

ROS-GSP4.1: 5′GCAGCTCAGCCAACTCTTTGTCTT

GAPDH-F: 5′TGGAAATCCCATCACcCATCT

GAPDH-R: 5′GTCTTCTGGGTGGCAGTGAT

### Constructs

The open reading frame of the *FIG-ROS(L)* and *FIG-ROS(S)* fusion gene was amplified by PCR from cDNA of ROS fusion positive patient tumors. These PCR products were cloned into the retroviral vector MSCV-Neo with a C-terminal Myc tag.

### Western blotting

Cells were lysed in 1× cell lysis buffer (Cell Signaling Technology) supplemented with Protease Arrest™ (G Biosciences) and separated by electrophoresis. All antibodies and reagents for immunoblotting were from Cell Signaling Technology.

### Soft agar assay and Xenograft

Retroviral transduced 3T3 cells were selected for G418 (0.5 mg/ml) for 7 days, and the cells were then cultured in soft agar in triplicate for 17 days. 1×10^6^ transduced 3T3 cells were resuspended in Matrigel (BD Biosciences) and injected subcutaneously at 2 sites into each nude mice. Each cell line was tested in 4 mice with a total of 8 injections. Mice were monitored daily for tumor formation and size, and were sacrificed when tumors reached approximately 1 cm×1 cm.

Approval for the use of animals in this study was granted by Cell Signaling Technology Animal Care and Use Committee with approval ID 650.

### *In vitro* kinase assay

Cell lysates from FIG-ROS transfected BaF3 cells were subjected to immunoprecipitation with Myc-Tag antibody, ROS immune complex were washed 3 times with cell lysis buffer, followed by kinase buffer (Cell Signaling Technology). Kinase reactions were initiated by re-suspending the ROS immune complex into 25 ul kinase buffer that contains 50 uM ATP, 0.2 uCi/ul [gamma32p] ATP, with 1 mg/ml of Poly (EY, 4∶1). Reactions were stopped by spotting reaction cocktail onto p81 filter papers. Samples were then washed and assayed for kinase activity by detection with a scintillation counter.

## Supporting Information

Figure S1 **Expression of ROS in primary cholangiocarcinoma samples.** (**A**) Detection of ROS expression by Western blot from protein lysates of a liver cancer patient (TC23). Arrows denote truncated forms of ROS. (**B**) Identification of genomic breakpoints of ROS fusions by sequencing genomic PCR products from two FIG-ROS positive patients (TC23 and TC03). U118MG was used as a control.
(TIF)

Figure S2 **Expression and characterization of ROS fusions in either 3T3 cells or BaF3 cells.** (**A**) Expression of FIG-ROS(L) and FIG-ROS(S) in 3T3 cells phosphorylate their downstream substrates, such as STAT3 and AKT. Arrow denote the correct size of FIG-ROS. (**B**) Expression of SLC34A2-ROS(S) in either 3T3 or BaF3 cells failed to activate its downstream signaling molecules. HCC78, which expresses SLC34A2-ROS(S), was included as a control. (**C**) Expression of FIG-ROS(L) and FIG-ROS(S) in BaF3 cells either in the presence of absence of IL3. (**D**) BaF3 cells were stably transfected with different ROS fusions, as well as empty Neo-Myc vector. BaF3 lysates were immunoprecipitated with Myc-tag antibody, and kinase assay was performed as described in experimental procedure. Kinase activity was expressed relative to that of empty Neo-Myc construct. Western blot showed similar amount of ROS proteins were used for kinase reaction.
(TIF)

Table S1 Phosphopeptides identified by LC-MS/MS in cholangiocarcinoma patient samples, as well as matching para-tumor samples. ‘y’ for phosphorylated tyrosine residue; ‘Y’ for unphosphorylated tyrosine residue.
(XLS)

Table S2 Total number of tyrosine phosphopeptides per protein identified by LC-MS/MS in cholangiocarcinoma patient samples, which is normalized against the number of total peptides from GSK3A (set to 100).
(XLS)
