# Supplementary figures and images for: Survey of Tyrosine Kinase Signaling Reveals ROS Kinase Fusions in Human Cholangiocarcinoma
Source: PLoS One. 2011 Jan 6;6(1):e15640. doi: 10.1371/journal.pone.0015640 (PMC3017127; doi:10.1371/journal.pone.0015640)

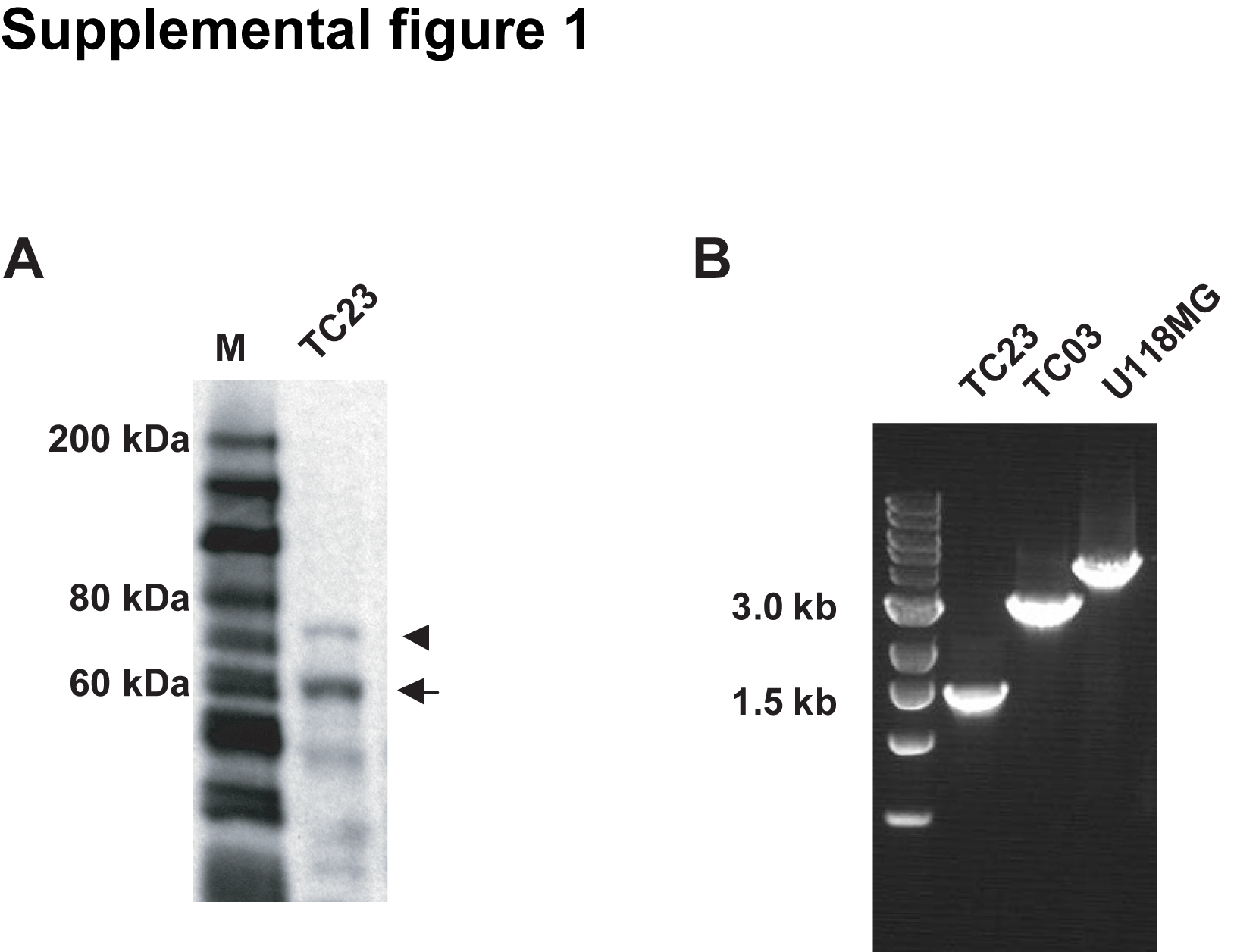

Supplement: Figure S1 — Expression of ROS in primary cholangiocarcinoma samples. (A) Detection of ROS expression by Western blot from protein lysates of a liver cancer patient (TC23). Arrows denote truncated forms of ROS. (B) Identification of genomic breakpoints of ROS fusions by sequencing genomic PCR products from two FIG-ROS positive patients (TC23 and TC03). U118MG was used as a control. (TIF) [file pone.0015640.s001.tif]

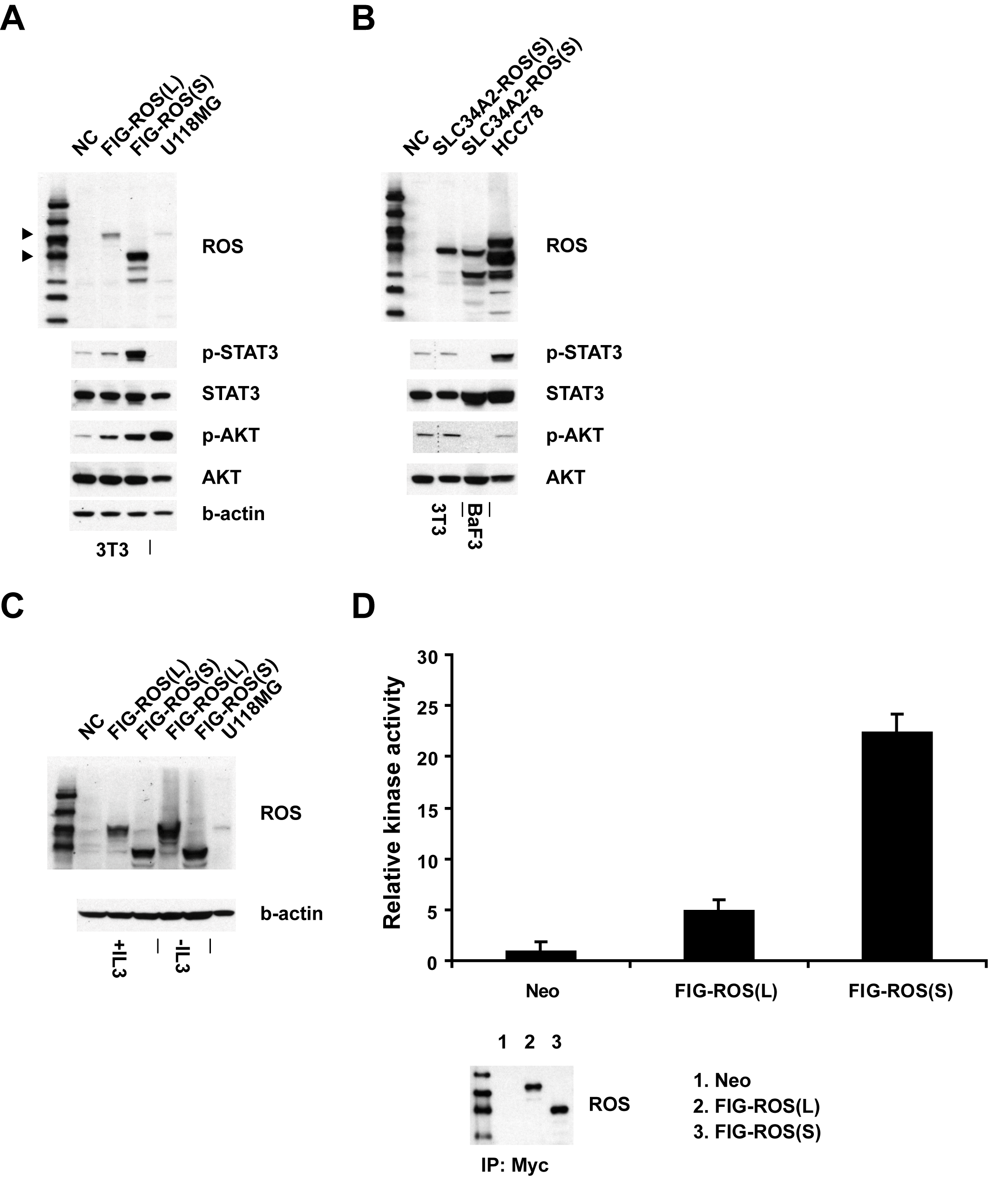

Supplement: Figure S2 — Expression and characterization of ROS fusions in either 3T3 cells or BaF3 cells. (A) Expression of FIG-ROS(L) and FIG-ROS(S) in 3T3 cells phosphorylate their downstream substrates, such as STAT3 and AKT. Arrow denote the correct size of FIG-ROS. (B) Expression of SLC34A2-ROS(S) in either 3T3 or BaF3 cells failed to activate its downstream signaling molecules. HCC78, which expresses SLC34A2-ROS(S), was included as a control. (C) Expression of FIG-ROS(L) and FIG-ROS(S) in BaF3 cells either in the presence of absence of IL3. (D) BaF3 cells were stably transfected with different ROS fusions, as well as empty Neo-Myc vector. BaF3 lysates were immunoprecipitated with Myc-tag antibody, and kinase assay was performed as described in experimental procedure. Kinase activity was expressed relative to that of empty Neo-Myc construct. Western blot showed similar amount of ROS proteins were used for kinase reaction. (TIF) [file pone.0015640.s002.tif]
